# Directed migration of cancer cells: a systems biophysics perspective

**DOI:** 10.3389/fcell.2026.1832990

**Published:** 2026-07-10

**Authors:** Andrew Mugler, Bumsoo Han

**Affiliations:** 1 Department of Physics and Astronomy, University of Pittsburgh, Pittsburgh, PA, United States; 2 Department of Mechanical Science and Engineering, Cancer Center at Illinois, Department of Bioengineering, Materials Research Laboratory, Institute of Genomic Biology and Beckman Institute, University of Illinois Urbana-Champaign, Urbana, IL, United States; 3 Biohub, Chicago, IL, United States

**Keywords:** chemotaxis, epidermal growth factor (EGF), rheotaxis, transforming growth factor beta (TGF-β), tumor microenvironment

## Abstract

Directed migration of cancer cells is crucial to tumor progression, including tumorigenesis and metastasis. During migration, cancer cells encounter complex environmental cues and must process these signals to determine their migration direction. These cues include chemical, mechanical, and fluidic signals that vary across space and time. Although cancer cell migration has been extensively studied and many molecular regulators and signaling pathways have been identified, this molecular knowledge alone is often insufficient to predict migratory behavior in the highly heterogeneous tumor microenvironment. In this perspective, we present a systems biophysics framework that views directed migration as an information-processing problem constrained by the physics of cellular motion. Within this framework, a migrating cancer cell can be conceptualized as an integrated system composed of interacting subsystems responsible for information acquisition, information processing, and actuation. These subsystems collectively enable cells to sense environmental cues, process competing signals, and generate directed movement. Using a combination of biophysical analysis, experiments, and theoretical modeling, we discuss recent efforts to quantify fundamental sensing limits, characterize individual and collective information acquisition, investigate the capacity of cellular signal-processing machinery, and identify physical constraints governing migration accuracy and persistence. We further describe how minimal theoretical models and reverse-engineering approaches can reproduce key features of cellular decision-making, including the use of logic-gate–like behavior to process multiple environmental cues. Together, these studies illustrate how systems-level biophysical analysis can provide predictive insights into how cancer cells navigate complex microenvironments and may help establish a unified framework for understanding directed migration across diverse cellular systems.

## Introduction

How cancer cells navigate the complex tumor microenvironment (TME) through directed migration is a central question in tumor progression and metastasis, underlying local invasion, intravasation, extravasation, and colonization (Worbs et al., 2017; Oudin and Weaver, 2017; Rou et al., 2011). During migration, cancer cells encounter complex environmental cues that vary across space and time, including chemical gradients (Rou et al., 2011; Moon et al., 2021), extracellular matrix (ECM) stiffness (DuChez et al., 2019; Moriyama et al., 2019; Ebata et al., 2020; Sunyer and Trepat, 2020; Shellar et al., 2021; Espina et al., 2022; Saez and Venturini, 2023), and interstitial fluid flow (Munson and Shieh, 2014; Lee et al., 2017; Valignat et al., 2013; Campinho et al., 2020; Chang et al., 2017; Shields et al., 2007). Cells must interpret these heterogeneous signals and determine a migration direction in order to navigate the highly heterogeneous TME effectively. Despite extensive research identifying molecular regulators and signaling pathways involved in cell migration, it remains difficult to predict migratory behavior in the highly complex environments that cancer cells experience *in vivo*.

A major challenge is that most studies have examined cell migration by focusing on individual molecular pathways or single environmental cues (Rou et al., 2011; SenGupta et al., 2021; Suhail et al., 2019; Welf et al., 2012; Devreotes and Horwitz, 2015). In contrast, cancer cells in tumors are simultaneously exposed to multiple interacting signals, many of which fluctuate dynamically and contain substantial noise. Understanding how cells process these complex inputs and translate them into directed motion therefore requires a framework that connects environmental sensing, intracellular signal processing, and mechanical actuation at the cellular scale. We propose that directed migration can be understood as an information-processing problem constrained by the physics of cellular motion. This perspective shifts the focus from individual molecular pathways to the physical principles that govern how cells acquire, process, and act upon environmental information. By treating directed migration as an information-processing system, we can identify fundamental limits and constraints that shape cellular behavior across diverse biological contexts. This approach complements molecular studies by providing a quantitative framework that links environmental signals to observable migration performance.

**FIGURE 1:**
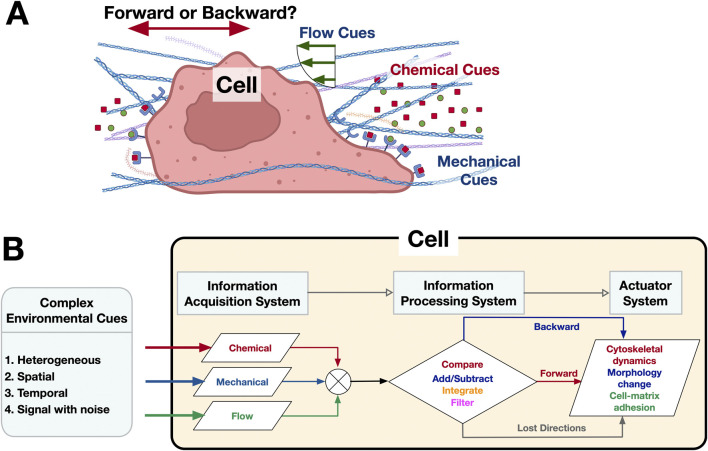
Systems biophysics framework for directed cancer cell migration. **(A)** Cancer cells in the tumor microenvironment are exposed to multiple environmental cues, including chemical gradients, mechanical signals from the extracellular matrix, and interstitial fluid flow. Cells process these cues to determine their migration direction. **(B)** Systems biophysics view of migrating cells as system of systems acquiring and processing information to determine migration direction. Directed migration emerges from the coordinated operation of these subsystems and is constrained by fundamental physical limits governing sensing accuracy, signal processing capacity, and mechanical force generation. Migration performance can therefore be analyzed in terms of measurable quantities such as directional accuracy, persistence, and migration speed.

In this perspective, we present a systems biophysics framework that conceptualizes directed migration as the coordinated operation of interacting cellular subsystems. Within this framework, a migrating cancer cell can be viewed as an integrated system that acquires environmental information, processes competing signals, and converts these signals into mechanical motion. As illustrated in Figure 1, directed migration emerges from the coordinated operation of three subsystems. The information acquisition subsystem determines how accurately cells measure environmental signals within the noisy TME, where stochastic molecular fluctuations and finite numbers of sensing molecules impose fundamental limits on sensing accuracy. The information processing subsystem interprets and prioritizes signals obtained from multiple environmental cues through intracellular signaling networks that compare signals across the cell body and determine the migration direction, while operating under finite molecular resources that constrain processing capacity. Finally, the actuator subsystem converts processed signals into cytoskeletal dynamics and mechanical forces that drive cell motion through coordinated regulation of actin polymerization, microtubule dynamics, and adhesion turnover. The performance of this subsystem is reflected in measurable migration properties such as directional accuracy, persistence, and migration speed.

By analyzing these subsystems within a unified systems biophysics framework, it becomes possible to connect environmental information acquisition, intracellular signal processing, and mechanical actuation in a quantitative manner. In the following sections, we examine how physical limits of sensing, constraints on signal-processing capacity, and biophysical constraints on cellular actuation together shape the performance of directed cancer cell migration.

## Information acquisition system

Within the systems biophysics framework, the information acquisition subsystem determines how accurately a cell can measure environmental signals before these signals are processed and translated into directed motion. Cancer cells acquire environmental information through molecular and mechanical sensing mechanisms that detect diverse cues in the TME. Chemical cues are sensed primarily through ligand-receptor interactions, in which chemokines or growth factors bind to cell-surface receptors and initiate intracellular signaling pathways that regulate directional migration (Rou et al., 2011; SenGupta et al., 2021; Suhail et al., 2019; Welf et al., 2012; Devreotes and Horwitz, 2015). Several soluble factors have been identified as chemoattractants for cancer cells, including epidermal growth factor (EGF), transforming growth factor-β (TGF-β), colony stimulating factor-1 (CSF1), CXCL12 (also known as SDF-1α), and CCL19. These signals typically activate receptor tyrosine kinases (RTKs) or G protein–coupled receptors (GPCRs), which initiate signaling cascades that ultimately regulate cytoskeletal dynamics and cell polarity (Rou et al., 2011; Van Haastert and Devreotes, 2004; Veltman et al., 2008).

In addition to chemical signals, cancer cells acquire information about the mechanical properties and architecture of the extracellular matrix (ECM). The ECM is a porous network composed primarily of collagen, elastin, and proteoglycans that provides structural support and adhesion sites necessary for cell migration (Ozcelikkale et al., 2017a). Through integrin-mediated adhesion and mechanosensitive signaling pathways, cells can detect ECM stiffness, topology, and fiber orientation (Paul et al., 2016; Griffith and Swartz, 2006; Baker and Chen, 2012; Polacheck et al., 2011; Rudzka and Olson, 2015). Mechanical gradients within the ECM can guide cell migration through mechanisms such as durotaxis, in which cells preferentially migrate toward stiffer regions of the matrix (Oudin and Weaver, 2017; Charras and Sahai, 2014; Wirtz et al., 2011). Furthermore, ECM architectures in tumor tissues are frequently reorganized by stromal cells such as cancer-associated fibroblasts, which can align collagen fibers and create confined migration tracks that facilitate cancer cell invasion (Rou et al., 2011; Paul et al., 2016).

Fluidic cues provide an additional source of environmental information (Polacheck et al., 2011). In many tumors, interstitial fluid pressure is elevated due to leaky vasculature and impaired lymphatic drainage associated with abnormal tumor growth and dense matrix deposition. These pressure gradients generate interstitial fluid flow through the ECM, which advects soluble signaling molecules produced by tumor and stromal cells. Cells can secrete and detect these molecules, a process known as autologous chemotaxis (Shields et al., 2007), and they can also mechanically detect the fluid flow itself (Polacheck et al., 2011; Polacheck et al., 2014). Mechanical detection occurs due to a tension imbalance in the cell membrane, caused by shear in liquid near surfaces or by pressure gradients in ECM (Polacheck et al., 2014). A tension imbalance imparts directional information via mechanosensitive ion channels on the cell surface (Bouffanais et al., 2013; Lee et al., 1999; Lombardi et al., 2008; Luchtefeld et al., 2024). Overall, migrating cancer cells encounter a dynamic environment in which chemical, mechanical, and fluidic cues coexist and fluctuate over time. The information acquisition subsystem must therefore detect relevant signals while operating in a noisy environment with substantial fluctuations in signal strength and direction.

### Physical limits of information acquisition

**FIGURE 2:**
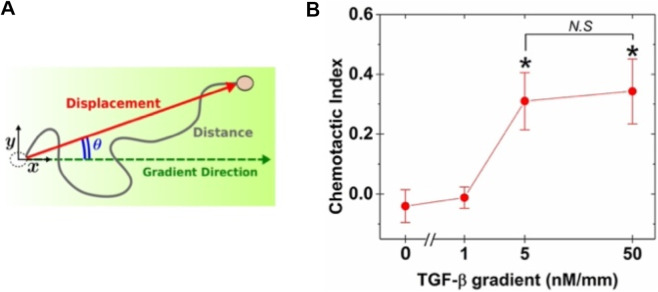
Physical sensing limit. **(A)** The chemotactic index is the cosine of the angle between the displacement direction and the gradient direction. **(B)** Chemotactic index of MDA-MB-231 breast cancer cell line in type I collagen over 9 h for four different TGF-β gradient strengths (data points indicate average and standard error of medians from three different experiments). Redrawn from (Varennes et al., 2019).

A fundamental question is how closely cancer cells operate near the physical limits of gradient detection previously derived for other cell types (Varennes et al., 2019). To address this question, we experimentally evaluated the chemotactic response of MDA-MB-231 breast cancer cells by measuring the chemotactic index, defined as the average cosine of the angle between the migration direction and the gradient direction (Figure 2A). As shown in Figure 2B, cells respond to gradients as shallow as g = 5 nM⋅mm^-1^, while the response saturates near 50 nM/mm.

The lower sensing threshold is comparable to that observed in other cell types known to operate near the fundamental limits of molecule counting (Ellison et al., 2016; van Haastert and Postma, 2007; Varennes et al., 2016). To illustrate this limit, consider a cell with characteristic width a ≈ 10 μm. For a gradient of g = 5 nM⋅mm^-1^, the concentration difference across the cell body is ga = 0.05 nM, corresponding to a relative change of ga/c ≈ 2%, where c = 2.5 nM is the background concentration. The number of attractant molecules occupying half of the cell volume is on the order of ca^3^ ≈ 1,500, implying that the concentration difference across the cell corresponds to approximately 30 molecules. In other words, cells detect gradients using only a few dozen molecules across their bodies. Similar estimates for epithelial cells in EGF gradients yield ga/c ≈ 6% and approximately 300 molecules across the cell body (Ellison et al., 2016).

These observations suggest that cancer cells operate close to the fundamental physical limits of gradient detection. The observed saturation at higher gradient strengths further indicates the presence of both lower and upper limits in the information acquisition capacity of the sensory machinery.

### Collective information acquisition in a noisy environment

In the tumor microenvironment, information acquisition often occurs not only at the level of individual cells but also through collective sensing by groups of cells. Collective migration is a key mechanism of cancer invasion and metastasis, in which groups of cells migrate coherently while maintaining intercellular contacts (Aceto et al., 2014; Cheung et al., 2013; Friedl et al., 2012; Puliafito et al., 2015). In such systems, cells may pool information through intercellular communication, potentially improving the accuracy of gradient detection.

**FIGURE 3:**
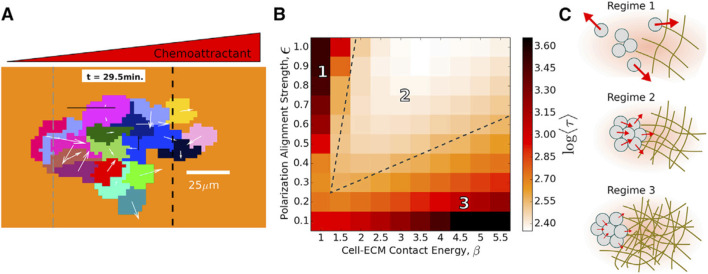
Collective sensing by cancer cells in a noisy environment. **(A)** Computational simulation of collective sensing and migration. Colors: individual cells; white arrows: polarization vectors. **(B)** Migration time as a function of cell-ECM adhesion energy β and polarization alignment strength ε. **(C)** Illustrations representing migratory behavior of clusters in respective regimes of parameter space from **(B)**. Larger values of ε correspond to larger cell polarization vectors (red arrows), whereas larger values of β correspond to an ECM that is more difficult to traverse. Redrawn from (Varennes et al., 2016).

Physical limits to collective gradient sensing have recently been derived theoretically (Ellison et al., 2016; Mugler et al., 2016). To investigate these effects, we developed a computational model of multicellular sensing and migration in which clusters of cells collectively measure noisy chemical gradients (Varennes et al., 2016), as illustrated in Figure 3A. The model incorporates stochastic receptor binding, intercellular communication, and fluctuating cell morphology, thereby coupling noisy sensing with collective migration dynamics. For simplicity it does not include persistent phenotypic differences between cells, such as mesenchymal, epithelial, or intermediate modes, which are known to occur in collective cancer cell migration and have been the subject of related theoretical work (Jolly et al., 2015; Sha et al., 2019). We focused on shallow-gradient regimes, where the concentration difference across a single cell is small relative to the background concentration. This regime is particularly relevant because experiments show that multicellular clusters can respond to gradients too shallow for individual cells to detect (Ellison et al., 2016; Malet-Engra et al., 2015; Rosoff et al., 2004).

Modeling these stochastic processes revealed several predictions. First, the fastest migration occurs when a balance is achieved between cellular polarization strength and adhesion to the extracellular matrix (Figure 3B). If polarization is too strong, cells may detach from the cluster and scatter, slowing net migration (Figure 3C, top). Conversely, excessive adhesion prevents cells from forming new protrusions, also slowing migration (Figure 3C, bottom). Optimal migration occurs between these extremes (Figure 3C, middle). Second, the model predicts the existence of an optimal cluster size for chemotaxis. Increasing cluster size enhances propulsive force but also increases spatial drag, and the optimal size reflects a tradeoff between these competing effects that depends on the strength of intercellular communication.

The model in Figure 3 is an example of emergent collective chemotaxis: cells collectively sense a chemical gradient and migrate together. In contrast, some cells exhibit individual-based collective chemotaxis (Kulesa and Fraser, 1998; Charteris and Khain, 2014), where agents migrate together but sense a gradient individually. By exploring both types of chemotaxis using mathematical modeling, we found that the precision of emergent chemotaxis is higher than that of individual-based chemotaxis for one-dimensional cell chains and two-dimensional cell sheets, but not three-dimensional cell clusters (Varennes et al., 2017). This result may help explain the migration of one-dimensional chains of cells (Cheung et al., 2013; Friedl et al., 2012) or two-dimensional cylindrical geometries of pancreatic duct cells during metastatic invasion (Bardeesy and DePinho, 2002).

### Information acquisition limits in cell decision-making

Cancer cells in the tumor microenvironment are frequently exposed to multiple competing cues simultaneously, such as chemical gradients, interstitial flow, and contact-mediated signals. We investigated the extent to which information acquisition precision alone determines which cue a cell follows under such conditions (González et al., 2025).

**FIGURE 4:**
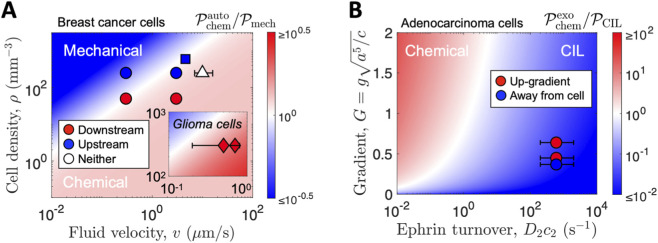
Sensory limits to cell decision-making. When two cues compete, we predict decision boundaries based on the physics of sensory precision (color maps) and compare to cancer cell migration data with no fitting (data points). **(A)** Cell responses to a chemical gradient vs. mechanical flow are consistent with the decision boundary. **(B)** In contrast, cell responses to a chemical gradient vs. contact inhibition of locomotion (CIL) are far from the decision boundary. This indicates that cells prioritize the gradient even when it is a weaker cue than CIL. Redrawn from (González et al., 2025).

Using physical arguments, we derived fundamental limits to sensing precision for three distinct cues: chemical gradients, mechanical flow, and contact inhibition of locomotion (CIL). These limits allow prediction of which cue should dominate when two cues compete, based solely on their relative sensing precision. Figure 4 illustrates these predictions as decision boundaries for two cue pairs: (A) chemical gradient versus mechanical flow and (B) chemical gradient versus CIL. When competing chemical gradients and mechanical flow are present, cellular behavior follows the predicted decision boundary. However, when chemical gradients compete with CIL, cells preferentially follow the chemical gradient even when its sensing precision is lower.

These observations indicate that cells are not passive sensors. Instead, intracellular signaling networks can amplify preferred cues and suppress competing signals. This finding highlights the importance of the next subsystem in the framework—the information processing subsystem, which determines how cells prioritize and interpret signals acquired from the environment.

## Information processing system

Within the systems biophysics framework, the information processing subsystem interprets signals acquired from the environment and determines the direction of cell migration. After environmental information has been obtained by the information acquisition subsystem, intracellular signaling networks must process these inputs, compare signals across the cell body, and convert them into intracellular instructions that guide cell polarity and motility. This processing occurs through interconnected signaling pathways that regulate cytoskeletal dynamics. Binding of soluble factors to their receptors or adhesion of integrins to the extracellular matrix activates intracellular signaling cascades that converge on small GTPases of the Rho family. These signaling pathways regulate actin polymerization, microtubule dynamics, and front–rear polarity, thereby linking signal processing to directional cell movement (Rou et al., 2011; Van Haastert and Devreotes, 2004; Veltman et al., 2008). Crosstalk between receptors and signaling pathways further modulates these responses, enabling cells to process signals from multiple environmental cues simultaneously (Hart et al., 2005; Lappano and Maggiolini, 2011; Shi and Chen, 2017).

From a systems perspective, this subsystem functions as the decision-making layer of the migration machinery. It must decode signals from multiple environmental inputs that may contain substantial noise, evaluate competing cues, and determine the direction of migration. Importantly, these computations are performed using a finite number of intracellular molecules, meaning that the capacity of cellular signaling networks to process information is inherently limited. These limits on signal-processing capacity can strongly influence how cells respond to multiple simultaneous cues in complex microenvironments.

### Signal processing capacity

To investigate how the capacity of intracellular signaling networks influences chemotactic behavior, we experimentally examined cancer cell migration in the presence of multiple chemoattractants. We found that the combination of transforming growth factor-β (TGF-β) and epidermal growth factor (EGF) suppresses the chemotactic performance of cancer cells (Moon et al., 2021). Each of these growth factors individually acts as a chemoattractant through its respective receptor and signaling pathway. However, when cells are exposed simultaneously to both gradients, the directional response is reduced.

**FIGURE 5:**
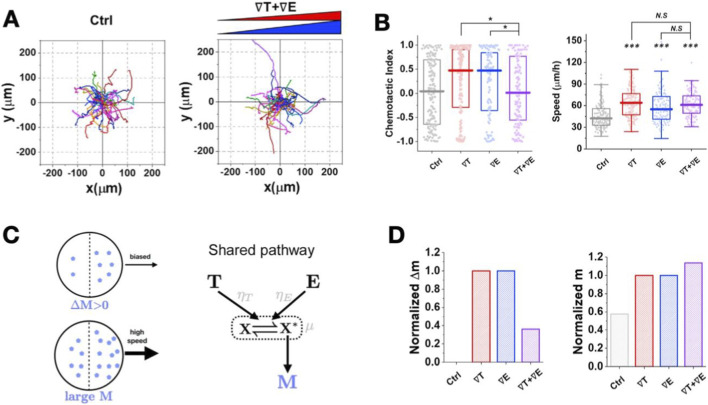
Signal processing capacity. **(A)** Cell trajectories of eKIC pancreatic cancer cell line in type I collagen over 3 h for the control case of no chemical cue (left) and for combined gradients of 50 nM⋅mm^-1^ TGF-β with 800 nM⋅mm^-1^ EGF (right). **(B)** Chemotactic index and speed distributions for the control, single-, and double-gradient cases. The chemotactic index shows antagonism: the response to both gradients is weaker than the response to either alone. **(C)** Mathematical model in which TGF-β and EGF convert a common component to its active state. Cell speed and chemotactic index scale with the concentration m of an intracellular species M, and its concentration difference Δm between the front and back of the cell, respectively. **(D)** The model reproduces the experimental observations, including antagonism of the chemotactic index. Redrawn from (Moon et al., 2021).

Using a microfluidic chemotaxis platform, we exposed breast cancer (MDA-MB-231) and pancreatic cancer (eKIC) cells to single or combined gradients of TGF-β (10 nM/mm) and EGF (200 nM/mm) and tracked their migration trajectories (Figure 5A). Cellular trajectories were analyzed to determine the chemotactic index (CI) and migration speed (Figure 5B). eKIC cells exhibited strong directional responses to both TGF-β and EGF gradients individually. However, under combined cues the chemotactic index was significantly reduced, indicating an antagonistic response. In contrast, migration speed increased under all chemical cues relative to control conditions but showed little difference between single and combined cues.

We further examined cell responses when a gradient was imposed on top of a uniform background concentration of chemoattractant, thereby increasing the total number of molecules present while maintaining the same gradient strength. Under these conditions, the chemotactic index was again significantly reduced while migration speed remained largely unchanged. These observations suggested that the suppression of directional migration might arise from physical limits in signal-processing capacity, rather than from biochemical mutual inhibition between signaling pathways.

To test this hypothesis, we developed a biophysical model in which antagonism arises from saturation of the signal processing machinery rather than repression of downstream pathways (Figure 5C). In the model, the migratory response is regulated by an internal molecular species M. Directed migration requires a difference in the concentration of M between the front and rear halves of the cell. This difference, Δm, serves as a proxy for the chemotactic index, while the average concentration m represents overall signaling activity and correlates with migration speed.

The model assumes that the signaling pathways activated by TGF-β (T) and EGF (E) converge onto a shared component X. Both cues catalyze the reversible conversion of X into an activated state X*, which in turn produces M. The dimensionless parameters $\eta_{T}$ and $\eta_{E}$ define the strengths of the catalysis reactions, and μ defines the degree to which the conversion reaction is unsaturated ( μ→0 ) or saturated ( μ→1 ). When both signals are present simultaneously, the shared pathway component can become saturated. In this regime, additional input signals no longer increase the front-rear difference in M, even though the total level of signaling activity remains high. The shared signaling component X in the model was formulated as a conceptual molecular entity rather than a specific biochemical species. This conceptual abstraction allows the framework to capture the effects of pathway convergence and saturation without assuming a particular molecular implementation. Nevertheless, several signaling hubs may serve as biological candidates for X, including shared downstream effectors of RTK and TGF-β signaling pathways such as PI3K, MAP kinase signaling components, or Rho-family GTPases including Rac1 and Cdc42 (Rou et al., 2011; Van Haastert and Devreotes, 2004; Veltman et al., 2008). Future studies combining molecular perturbations with quantitative migration measurements may help identify which signaling nodes function as capacity-limiting resources during multi-cue processing.

Mathematical analysis of this shared-pathway model reproduces the experimentally observed behavior: antagonism in the chemotactic index (Δm) but not in migration speed (m) (Figure 5D). As the pool of X molecules becomes fully converted to the activated state X*, the spatial difference in signaling between the front and back of the cell diminishes, reducing directional sensing. However, the total concentration of M remains elevated, maintaining migration speed. These results support the hypothesis that saturation of shared signaling components imposes a fundamental limit on the capacity of cells to process multiple environmental cues simultaneously.

### The processing system as a logic gate for migration decisions

We further investigated how the cellular information processing subsystem operates when cancer cells are exposed simultaneously to chemical and fluidic cues. Compared with two chemical cues, the combination of chemical and fluidic signals introduces additional complexity because fluid flow not only acts as a directional cue but also advects soluble molecules, thereby modifying the extracellular concentration gradient. To understand how cells process such coupled signals, we reverse-engineered the cellular processing system and constructed a minimal functional framework capable of reproducing the migration behavior observed under combined chemical and fluidic cues (Moon et al., 2023).

**FIGURE 6:**
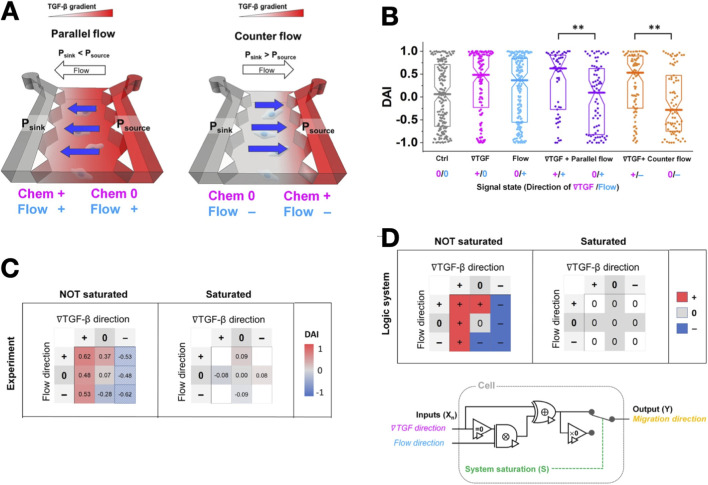
Cell signal processing system as a logic gate. **(A)** Schematic description of a microfluidic platform to induce the chemical gradient with the pressure-driven flow (Flow). Flow direction is defined based on the chemical gradient–the chemical gradient and pressure gradient are aligned (parallel flow), or the chemical gradient and pressure gradient are in opposing directions (counter flow). **(B)** DAI distributions of all collected trajectories of eKIC cells with respect to each signal state of ∇TGF (magenta)/flow direction (dark cyan). Seven signal states are presented, where 0/+ in flow indicates no-∇TGF whereas 0/+ in parallel flow indicates too shallow ∇TGF for cells to detect. Box: quartiles with a median line in the middle of the box. Dot: a DAI from a single trajectory; cell trajectories N > 50. **: p < 0.01, (Mann–Whitney test). **(C)** Heat map for experimental results of DAI medians of not saturated (left) and saturated (right) cases. The system saturation is considered with experimental groups of the higher TGF-β background noise (TGF = 10 nM). **(D)** The truth table of the ternary logic system with signal states (+, 0, and −). The proposed ternary logic gate model of cell signal processing system. Redrawn from (Moon et al., 2023).

Using a microfluidic platform, we exposed a murine pancreatic cancer cell line (eKIC) to gradients of TGF-β in the presence of controlled interstitial flow (Figure 6A). Note that eKIC cells are not known to secrete and bind molecules to detect flow (autologous chemotaxis) and are therefore presumed to detect flow via mechanotransduction. The concentration gradient of TGF-β was significantly altered by the presence and direction of fluid flow. When interstitial flow was imposed parallel to the chemical gradient (meaning upstream and up-gradient are parallel), the gradient became shallow across most of the channel except near the sink region. As a result, cells near the sink experienced both positive chemical and positive fluidic cues (”+/+” state), whereas cells near the source experienced only a positive fluidic cue and effectively no detectable chemical cue (”0/+” state). Here, the sign of the cues means whether up-gradient/upstream points right (+) or left (−), and the magnitude (zero or nonzero) of the chemical cue was determined based on whether the gradient magnitude exceeded the previously established physical sensing limit (Ellison et al., 2016; Varennes and Mugler, 2016). In contrast, when counter flow was imposed, the upstream and up-gradient directions were opposite. Under these conditions, the gradient became steep primarily near the source region. Cells near the sink experienced no detectable chemical gradient and a negative fluidic cue (”0/−” state), whereas cells near the source were exposed to two competing signals: a positive chemical gradient and a negative fluidic cue (”+/−” state).

Migration behavior was quantified using the directional accuracy index (DAI), defined as the average cosine of the angle between cell displacement and the direction of the gradient or upstream flow (Figure 6B). Under single-cue conditions, cells exhibited directional bias toward the respective cue: TGF-β gradients or fluid flow. In the parallel-flow configuration, directional accuracy was significantly enhanced in the “+/+” region near the sink. However, cells near the source under the “0/+” condition lost directional accuracy despite the presence of flow. In the counter-flow configuration, cells in the “+/−” state remained biased toward the chemical gradient, indicating that the chemical cue dominated the decision-making process even when opposed by fluid flow. Conversely, cells in the “0/−” state responded primarily to the fluidic cue. These observations suggest that cells respond to fluid flow only when the chemical gradient falls below the sensory detection threshold.

To summarize these behaviors, we compiled all experimental conditions into a heat map representing migration responses under each signal state (Figure 6C). The heat map reveals a hierarchy in cue prioritization: when a detectable chemical gradient is present, cells preferentially migrate along that gradient regardless of the fluidic cue. An important observation arises in the “0/+” state, which occurs in two experimentally distinct scenarios: flow-only conditions and parallel-flow conditions with high background TGF-β concentration. In the latter case, although the chemical gradient is effectively absent, the high background concentration produces strong receptor occupancy, corresponding to saturation of the signal-processing system. Under such saturation, cells fail to respond to fluidic cues as well.

Based on these observations, we propose that the cellular processing system can be conceptualized as a ternary logic gate that integrates chemical and fluidic inputs (Figure 6D). In this framework, chemical and fluidic cues are represented as discrete signal states (+, 0, −). Logical operators describe how these signals are combined to determine the migration direction. The monadic operator “ = 0”returns + output to 0 input but 0 output otherwise. Another monadic operator, “×0,” returns all zeros regardless of the input states. We also used dyadic operators represented as ⊗ and ⊕, which simply multiply and add two inputs to return the corresponding outputs, respectively. When the signaling system is not saturated (S = 0), cells preferentially migrate along the TGF-β gradient regardless of the presence of fluid flow, corresponding to a selection gate that prioritizes the chemical cue. In contrast, when the system becomes saturated (S = 1), the circuit returns a zero output, preventing directional migration despite the presence of external signals. This conceptual framework provides a simplified representation of how cancer cells process multiple environmental cues while avoiding misguided migration when the signal-processing capacity of the cell is exceeded.

The output of this information processing subsystem ultimately regulates cytoskeletal dynamics and force generation that produce directed cell movement, which we describe next as the actuator subsystem.

## Actuator system

Within the systems biophysics framework, the actuator subsystem converts processed signals into the mechanical motion that drives directed cell migration. After environmental cues are acquired and processed, intracellular signaling networks activate molecular machinery that reorganizes the cytoskeleton and generates the forces required for cell movement. This subsystem consists of coordinated molecular processes that regulate actin polymerization, microtubule dynamics, and adhesion turnover. Signaling pathways discussed previously, including those involving PI3K and Rho-family GTPases, regulate these cytoskeletal activities and establish front–rear polarity that guides directional movement (Espina et al., 2022). Through these mechanisms, the actuator subsystem translates intracellular signaling outputs into mechanical forces that propel the cell through the surrounding extracellular matrix.

Cell migration is driven primarily by actin-based protrusion at the leading edge, contraction of the actomyosin network, and formation and release of adhesive contacts with the extracellular matrix. Actin polymerization generates protrusive structures such as lamellipodia and filopodia, which extend the cell membrane in the direction of migration. Integrin-mediated adhesions anchor the cell to the extracellular matrix, allowing traction forces to be transmitted through the cytoskeleton. At the rear of the cell, actomyosin contraction promotes detachment and forward movement of the cell body. The coordinated regulation of these processes enables the cell to generate persistent and directional motion.

### Physical constraints between accuracy and persistence

The performance of the actuator subsystem can be characterized by three measurable properties of cell migration: directional accuracy, persistence, and speed. Directional accuracy reflects how closely the cell moves along the true gradient direction. Persistence describes the tendency of a cell to maintain its current direction of motion rather than frequently changing direction. Speed represents the rate at which the cell moves through its environment. High migration performance would ideally involve high accuracy, strong persistence, and rapid movement.

**FIGURE 7:**
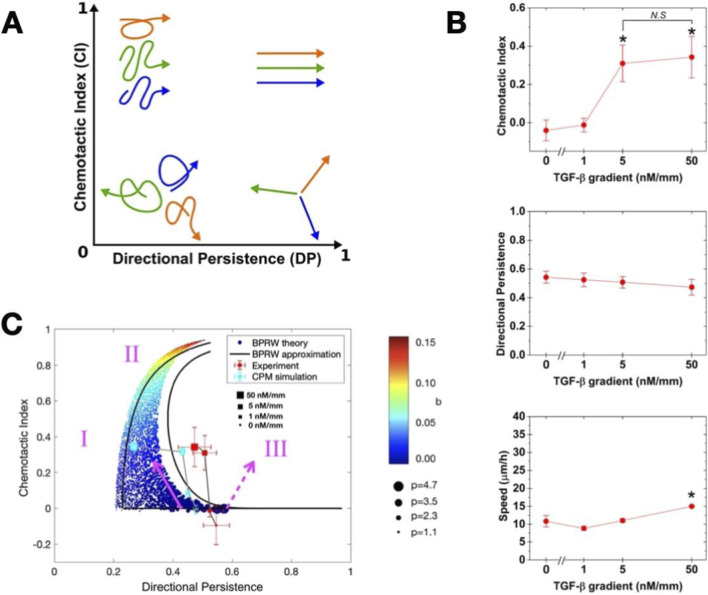
Physical constraints of cellular actuator system under direct migration. **(A)** Illustration of example cell trajectories in the space of accuracy, quantified by the chemotactic index, and persistence, quantified by the directional persistence index (displacement over total distance). **(B)** Chemotactic index, directional persistence, and speed of MDA-MB-231 breast cancer cell line in type I collagen over 9 h for four different TGF-β gradient strengths (data points indicate average and standard error of medians from three different experiments). **(C)** Theory (colored circles), approximations (black lines), simulation data (cyan squares), and experimental data (red squares) in the accuracy-persistence space. Magenta numerals: “forbidden” regions. Magenta arrows: paths allowed (solid) and disallowed (dashed) by the theory. Redrawn from (Varennes et al., 2019).

However, the relationships among these quantities are not fully understood. In particular, it remains unclear whether improving one property necessarily constrains the others. For example, cells may move accurately but with low persistence, or persistently but with poor directional accuracy. These possibilities are illustrated schematically in Figure 7A. To investigate whether these migration properties are physically constrained, we analyzed the behavior of cells undergoing chemotaxis in TGF-β gradients (Varennes et al., 2019), as shown in Figure 7B. As the gradient strength increased, the chemotactic index, which measures directional accuracy, increased significantly. In contrast, directional persistence decreased slightly while migration speed increased modestly. These results indicate that chemical gradients primarily enhance directional bias without strongly altering persistence or speed. Computational simulations similar to those used in Figure 3A, but applied to single-cell migration, reproduced these trends. The simulations suggest that the mechanisms governing chemotactic accuracy and directional persistence are largely decoupled within the cell’s internal regulatory network. This observation is consistent with the view that directional sensing and polarity formation represent distinct but interacting components of the migration machinery (Shi et al., 2013).

To further examine the relationships among chemotactic index, speed, and persistence, we developed a migration model based on the biased persistent random walk framework (Alt, 1980; Othmer et al., 1988). Under general assumptions, this model predicts that the chemotactic index and persistence are constrained within a characteristic crescent-shaped region in parameter space, as illustrated in Figure 7C. Increasing the directional bias parameter, analogous to strengthening cell polarization, raises the chemotactic index while having only a limited effect on persistence. Conversely, increasing persistence is possible only at either low or high values of the chemotactic index. These results indicate that migration accuracy and persistence are not independent but are constrained by fundamental physical relationships governing stochastic cell motion. As a result, entire regions of the accuracy-persistence space are inaccessible to migrating cells such as Region I, II and III. These constraints help qualitatively explain experimental observations showing that chemical gradients substantially increase directional accuracy while producing relatively modest changes in persistence (the quantitative disagreement reflects the extreme simplicity of the biased persistent random walk model). More broadly, they illustrate how the actuator subsystem operates under biophysical limits that shape the achievable performance of directed cell migration.

Together with the information acquisition and information processing subsystems described above, the actuator subsystem completes the systems-level framework governing directed cancer cell migration. In addition, the actuator system may involve feedback from the ECM remodeled by migrating cells. Migrating cells within a 3D ECM actively remodel their surroundings through matrix degradation, force-mediated fiber reorganization, and interactions with stromal cells such as cancer-associated fibroblasts (Oudin and Weaver, 2017; Ozcelikkale et al., 2017b). As a result, cells do not simply respond to pre-existing environmental cues but can also modify the very signals that guide subsequent migration. This bidirectional coupling introduces a dynamic feedback loop between cellular migration and the local microenvironment. ECM remodeling can also alter the structural architecture of the matrix, generating aligned fibers and confined migration tracks that influence cell polarity, directional persistence, and migration mode. Such confinement may modify the physical constraints governing migration performance, including the relationship between directional accuracy and persistence. Because ECM remodeling often occurs on timescales that differ from those of mechanosensing and directional decision-making, understanding how dynamic matrix remodeling and geometric confinement interact with information acquisition and actuation remains an important challenge for future systems-level studies of directed migration.

## Summary

In this perspective, we present a systems biophysics framework for understanding directed migration of cancer cells. In this view, a migrating cell functions as an integrated system composed of interacting subsystems responsible for information acquisition, information processing, and actuation. Environmental cues in the tumor microenvironment provide information that cells must acquire and process before converting the resulting signals into mechanical motion. Directed migration therefore emerges from the coordinated operation of these subsystems under fundamental physical constraints.

Within this framework, the information acquisition subsystem determines how accurately cells measure environmental signals such as chemical gradients, extracellular matrix mechanics, and interstitial fluid flow. Physical limits arising from stochastic molecular fluctuations constrain the accuracy of gradient detection at both the single-cell and collective levels. The information processing subsystem interprets signals from multiple environmental cues and determines migration direction through intracellular signaling networks. Because these networks operate with finite molecular resources, their signal-processing capacity can become saturated, limiting how cells respond to multiple simultaneous cues. The actuator subsystem converts processed signals into cytoskeletal dynamics and mechanical forces that drive cell motion. The resulting migration behavior can be characterized by measurable quantities such as directional accuracy, persistence, and migration speed, which are themselves constrained by fundamental physical relationships governing stochastic cell movement.

By examining these subsystems within a unified systems framework, it becomes possible to connect environmental sensing, intracellular signaling, and mechanical motion in a quantitative manner. This perspective complements traditional molecular studies by emphasizing the physical principles that constrain how cells acquire, process, and act upon environmental information. As a result, systems-level biophysical analysis can provide predictive insight into how cancer cells navigate complex microenvironments. More broadly, the systems biophysics framework presented here may help bridge the disciplines of systems biology and biological physics. Systems biology focuses on identifying interacting components within biological networks, whereas biological physics emphasizes how physical laws constrain biological behavior. By combining these perspectives, the systems approach outlined here provides a quantitative framework for understanding directed migration that can generate experimentally testable predictions.

Although this perspective focuses on cancer cell migration, the principles described here are likely applicable to other cellular systems in which cells navigate complex environments. Similar mechanisms may govern endothelial cell migration during angiogenesis, immune cell trafficking during inflammation and anti-tumor responses, and other forms of directed cell movement. More generally, applying systems biophysics approaches to cellular behaviors may reveal unifying principles that govern how living systems sense, process, and respond to complex environmental information. An important future direction will be to systematically evaluate information acquisition, information processing, and actuator behavior within a unified experimental platform, enabling direct assessment of the extent to which the systems-level principles described here are conserved across different cellular contexts. Ultimately, viewing directed cell migration through a systems biophysics framework may help transform complex cellular behaviors into quantitatively predictable biological processes.
